# Beyond the infection: mapping the risk of cardiovascular events post-scrub typhus in a nationwide cohort study

**DOI:** 10.1080/22221751.2025.2467766

**Published:** 2025-02-13

**Authors:** Jih-Kai Yeh, Victor Chien-Chia Wu, Shao-Wei Chen, Chia-Ling Wu, Yu-Sheng Lin, Chun-Wen Cheng, Chih-Hsiang Chang, Michael Wu, Pao-Hsien Chu, Shang-Hung Chang, Yu-Tung Huang

**Affiliations:** a Division of Cardiology, Chang Gung Memorial Hospital, Linkou Medical Center, Taoyuan City, Taiwan; b Department of Cardiothoracic and Vascular Surgery, Chang Gung Memorial Hospital, Linkou Medical Center, Taoyuan City, Taiwan; c Center for Big Data Analytics and Statistics, Chang Gung Memorial Hospital, Linkou Medical Center, Taoyuan City, Taiwan; d Department of Cardiology, Chang Gung Memorial Hospital, Chiayi, Taiwan; e Department of Infectious Diseases, Chang Gung Memorial Hospital, Linkou Medical Center, Taoyuan City, Taiwan; f Department of Nephrology, Kidney Research Center, Chang Gung Memorial Hospital, Linkou Medical Center, Taoyuan City, Taiwan; g Divison of Cardiovascular Medicine, Arrhythmia Services Section, Rhode Island Hospital, Warren Alpert School of Medicine, Brown University, Providence, USA; h Graduate Institute of Nursing, Chang Gung University of Science and Technology, Taoyuan City, Taiwan

**Keywords:** Scrub typhus, *Orientia tsutsugamushi*, cardiovascular risk, heart failure hospitalization, atrial fibrillation

## Abstract

Scrub typhus, caused by *Orientia tsutsugamushi*, often involves multiple organs, but its cardiovascular (CV) sequelae in survivors remain under-researched. This retrospective cohort study analyzed data from the National Health Insurance Research Database (NHIRD) spanning 2010–2015 to assess CV risks among scrub typhus survivors. Excluding those with prior CV events, we focused on outcomes such as acute myocardial infarction (AMI), heart failure hospitalization (HFH), strokes, new-onset atrial fibrillation (AF), aortic aneurysm or dissection, venous thromboembolism (VTE), and CV death. From 2,269 scrub typhus patients without previous CV events (mean age 47.8 ± 16.1; 38.0% female), and a matched control group (*n* = 2,264), we observed a higher incidence of HFH, new-onset AF, and total CV events in the scrub typhus cohort. Adjusted hazard ratios (aHRs) were 1.97 (95% CI: 1.13–3.42) for HFH, 2.48 (95% CI: 1.23–5.0) for new-onset AF, and 1.43 (95% CI: 1.08–1.91) for total CV events. Other outcomes did not significantly differ. Scrub typhus survivors exhibit an increased risk of CV events, particularly HFH and new-onset AF, underscoring the importance of heightened physician awareness and post-infection cardiac surveillance.

## Background

Scrub typhus is rickettsial infection caused by *Orientia tsutsugamushi*, endemic to Asia-Pacific regions [1]. Clinical presentations range from nonspecific symptoms to life-threatening complications [2]. Severe cases of scrub typhus tend to occur in the elderly, in individuals with a prolonged duration of illness (greater than 7 days), and in those without the pathognomonic eschar [3]. The diagnosis of scrub typhus relies heavily on clinical suspicion, particularly in patients with a history of exposure in endemic regions [4,5].

The pathophysiological findings in scrub typhus include widespread vasculitis and perivascular inflammation, in the affected organs [6]. Historical investigations during and after World War II highlighted cardiovascular (CV) disturbances associated with scrub typhus, including myocardial involvement and arrhythmias. Foundational studies by Sokolow and Garland [7], Levine [8], and Likoff [9] detailed these complications, emphasizing their clinical importance. In modern cohort studies, cardiac involvement in scrub typhus infection is common, with reduced left ventricular ejection fraction observed in 31%–43% of patients and elevated cardiac troponin T levels in 62%–73% of patients [10,11]. Abnormal electrocardiogram (ECG) findings, such as ST-segment changes, T wave inversion, and QT interval prolongation, correlate with infection severity and myocardial injury due to active inflammation in the myocardium [12,13].

Although patients with milder disease recover within 48 h of initiating appropriate antibiotics, subclinical structural and functional impairments in the CV system may go unrecognized. Comprehensive research on the subsequent development of CV diseases in patients with scrub typhus infection is currently unavailable. Therefore, a longitudinal cohort study was conducted to investigate whether patients with scrub typhus have an increased risk for major CV events.

## Methods

### Data source

We utilized data from the National Health Insurance Research Database (NHIRD) and the Notifiable Infectious Disease (NID) surveillance reporting system maintained by the Taiwan Centers for Disease Control (CDC) center. The NHIRD compiles comprehensive records of inpatient, outpatient, and emergency services, including diagnoses, prescriptions, examinations, operations, and related expenditures from healthcare providers. The database encompasses over 99% of the 23 million Taiwanese population.

Scrub typhus has been a NID in Taiwan since 1955, requiring mandatory reporting of suspected cases to Taiwan CDC centers. Diagnostic confirmation is performed at the Taiwan CDC Vector-borne Viral and Rickettsial Diseases Laboratory in Taipei. Serological testing using indirect immunofluorescent assay serves as the primary diagnostic method, with confirmation based on an IgM titer of 1:80, an IgG titer of 1:320, or a fourfold increase in IgG between acute and convalescent paired sera. Since 2006, molecular diagnostics utilizing real-time polymerase chain reaction have been introduced to enhance sensitivity. Additionally, blood samples collected during the acute phase are routinely used for isolation of *Orientia tsutsugamushi* [14].

Data linkage between the NHIRD and NID was facilitated by the Health and Welfare Data Center (HWDC). To ensure patient privacy, all data from the NHIRD and NID databases were anonymized and accessed for research purposes at HWDC sites.

### Identification of study cohort

Patients aged ≥20 years diagnosed with scrub typhus between 2010 to 2015 were included (*n* = 2,347). The study index date was the date of the first diagnosis in outpatient or discharge records. Patients with prior major CV events before the index date were excluded (*n* = 78). Major CV events were defined as myocardial infarction, heart failure, ischemic or hemorrhagic stroke, atrial fibrillation, aortic aneurysm or dissection, and venous thromboembolism.

**Figure 1.**
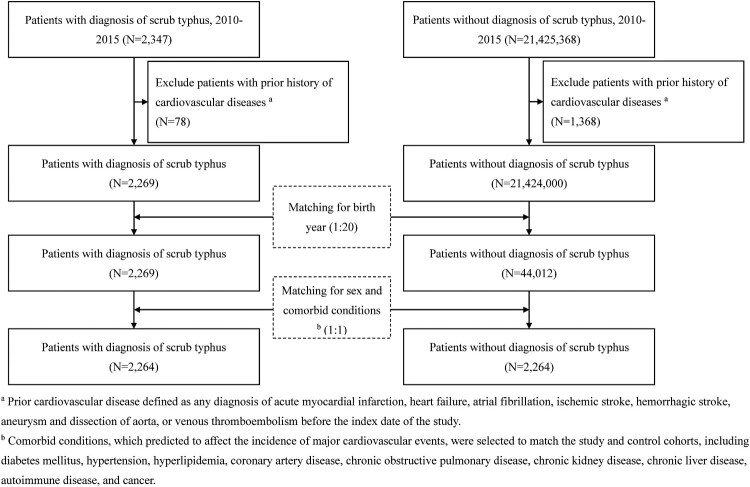
Flow chart of selection and inclusion of patients with scrub typhus and the control population.

The comparison cohort was drawn from the general population, excluding individuals with any prior scrub typhus diagnosis or major CV events before 2010 (*n* = 21,424,000). Each scrub typhus case was matched to a control based on age, gender, and comorbidities. A two-step frequency matching was utilized: first, a 1:20 ratio to select potential controls by birth year, and subsequently, a 1:1 ratio matching cases to controls based on age, sex, and comorbid conditions, including diabetes mellitus, hypertension, hyperlipidemia, coronary artery disease (CAD), chronic obstructive pulmonary disease (COPD), chronic kidney disease (CKD), chronic liver disease, autoimmune disease, and cancer. The yielded 2,264 scrub typhus patient and 2,264 matched controls. The cohort identification process is depicted in Figure 1.

### Measurement of covariates and study outcomes

Covariates included common CV risk factors: age, gender, diabetes mellitus, hypertension, hyperlipidemia, CAD, COPD, CKD, chronic liver disease, autoimmune disease, and cancer. Diagnoses were identified using ICD-9-CM codes prior to 2016 and ICD-10-CM codes, thereafter, requiring at least two outpatient diagnoses or one primary discharge diagnosis in the year preceding the index date.

CV outcomes of interest were acute myocardial infarction (AMI), heart failure hospitalization (HFH), ischemic stroke, hemorrhagic stroke, new-onset atrial fibrillation (AF), aneurysm or dissection of aorta, venous thromboembolism (VTE), and CV death. Follow-up extended from the index date to the first CV event, death, insurance withdrawal, or until December 31, 2017. Disease codes for all outcomes are provided in Supplementary Table 1.

### Statistical analysis

Frequency matching was employed to balance baseline covariates between the scrub typhus and control group. Baseline differences were assessed using absolute standardized mean differences (SMDs), with an SMD < 0.1 indicating a balanced distribution of covariates. Kaplan-Meier survival analysis was used to compare cumulative probabilities of CV events between cohorts, with statistical significance determined via log-rank tests.

Incidence rate ratios (IRRs) and their 95% confidence intervals (CIs) were calculated for each CV outcome using Poisson regression. To evaluate the association between scrub typhus and CV outcomes, Cox proportional hazards regression models were applied to estimate hazard ratios (HRs) with 95% CIs for each event. Adjustments were made for key covariates, including age, gender, and comorbidities.

Subgroup analyses were conducted to explore the effects of age, gender, and pre-existing comorbidities on specific CV events. All statistical tests were two-sided, with a *p*-value of less than 0.05 considered statistical significance. No adjustments were made for multiple comparisons. Statistical analyses were performed using SAS software (Version 9.4; SAS Institute Inc., Cary, NC).

### Ethics statement

The Chang Gung Medical Foundation's Institutional Review Board approved the study protocol (IRB No: 201901517B0). All data were anonymized and analyzed on-site at HWDC, in accordance with the Declaration of Helsinki and the Declaration of Taipei regarding ethical considerations for health databases by the World Medical Association.

## Results

### Subject characteristics

The study cohort comprised 2,269 patients diagnosed with scrub typhus, with a mean age of 47.8 ± 16.1 years; 38.0% were female. The prevalence of comorbid conditions in this cohort was as follows: diabetes mellitus (7.1%), hypertension (14.3%), COPD (2.6%), CKD (0.3%), autoimmune disease (0.4%), and cancer (2.6%). After matching the study cohort 1:1 with the comparison cohort for age, gender, and comorbidities, the cohorts (n = 2264 for each) showed a balanced distribution of these variables, with all absolute SMD < 0.01. Table 1 details the baseline characteristics of the cohorts, both with and without scrub typhus, before and after matching.

**Table 1.** Baseline characteristics of study patients with Scrub typhus or without Scrub typhus.

	Baseline			After 1:1 frequency matching		
	Scrub typhus	Control		Scrub typhus	Control	
Characteristics	*N* (%)	*N* (%)	abs SMD	*N* (%)	*N* (%)	abs SMD
Total	2,269	44,012		2,264	2,264	
Age (mean ± SD)	47.8 ± 16.1	47.3 ± 15.9	0.033	47.8 ± 16.0	48.0 ± 16.0	0.013
Age						
20–65 years	1965 (86.6)	38587 (87.7)	0.032	1962 (86.7)	1955 (86.4)	0.009
>65 years	304 (13.4)	5425 (12.3)	0.032	302 (13.3)	309 (13.7)	0.009
Sex						
Male	1408 (62.1)	21869 (49.7)	0.251	1405 (62.1)	1405 (62.1)	0.000
Female	861 (38.0)	22143 (50.3)	0.251	859 (37.9)	859 (37.9)	0.000
Comorbid conditions						
Diabetes mellitus	161 (7.1)	2497 (5.7)	0.059	158 (7.0)	158 (7.0)	0.000
Hypertension	324 (14.3)	4659 (10.6)	0.112	322 (14.2)	322 (14.2)	0.000
Hyperlipidemia	30 (1.3)	423 (1.0)	0.034	30 (1.3)	30 (1.3)	0.000
CAD	28 (1.2)	411 (0.9)	0.029	25 (1.1)	25 (1.1)	0.000
COPD	60 (2.6)	858 (2.0)	0.046	55 (2.4)	55 (2.4)	0.000
CKD	6 (0.3)	235 (0.5)	0.043	4 (0.2)	4 (0.2)	0.000
Chronic liver disease	55 (2.4)	782 (1.8)	0.045	52 (2.3)	52 (2.3)	0.000
Autoimmune disease	10 (0.4)	230 (0.5)	0.012	10 (0.4)	10 (0.4)	0.000
Cancer	59 (2.6)	1677 (3.8)	0.069	58 (2.6)	58 (2.6)	0.000

^a^ CAD: coronary artery disease; CKD: chronic kidney disease; COPD: chronic pulmonary obstructive disease; SD: standard deviation.

### Long-term cardiovascular events

**Figure 2.**
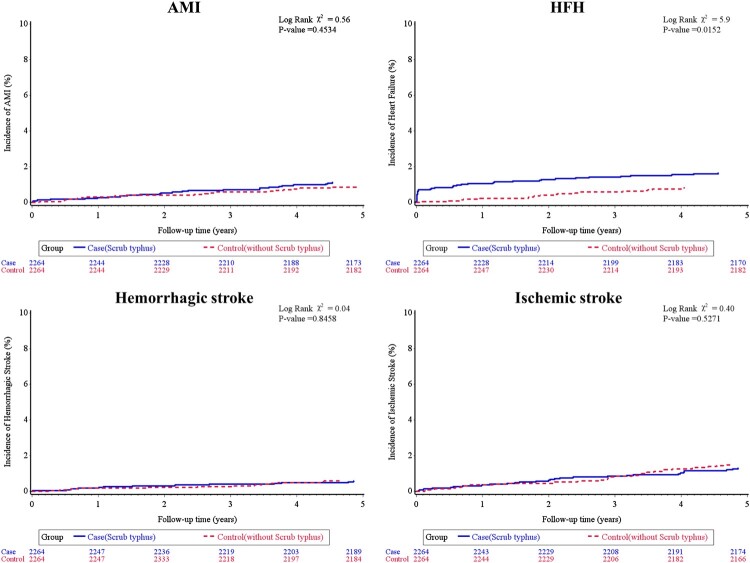
Cardiovascular outcomes in people with or without scrub typhus infection. Shown are the cumulative incidence of cardiovascular events in patients with scrub typhus infection, as compared with control individuals, on the outcome of acute myocardial infarction (Panel A); the outcome of heart failure hospitalization (Panel B); the outcome of hemorrhagic stroke (Panel C); the outcome of ischemic stroke (Panel D). *P* values were estimated with the use of Cox regression models.

**Figure 3.**
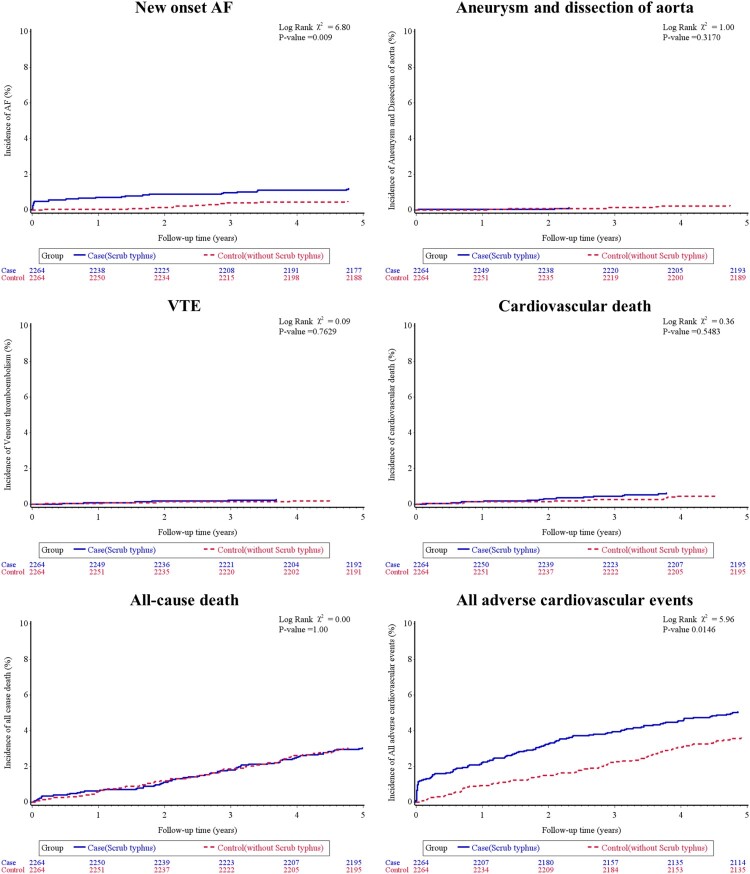
Cardiovascular outcomes in people with or without scrub typhus infection. Shown are the cumulative incidence of cardiovascular events in patients with scrub typhus infection, as compared with control individuals, on the outcome of new-onset atrial fibrillation (Panel A); the outcome of aneurysm and dissection of aorta (Panel B); the outcome of venous thromboembolism (Panel C); the outcome of cardiovascular death (Panel D); the outcome of all-cause death (Panel E); and the outcome of all major cardiovascular events (Panel F). All relevant adverse cardiovascular events composite of acute myocardial infarction, heart failure hospitalization, ischemic stroke, hemorrhagic stroke, new-onset atrial fibrillation, aortic disease, venous thromboembolism, or cardiovascular death. *P* values were estimated with the use of Cox regression models.

**Figure 4.**
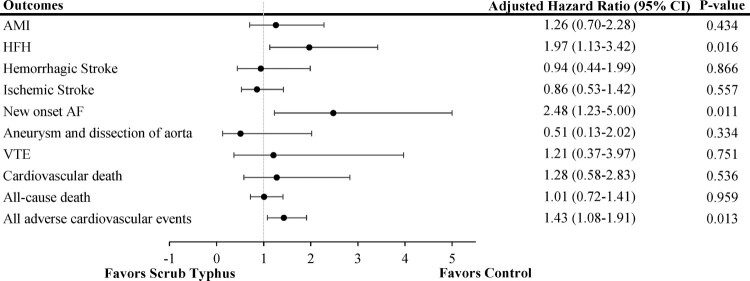
Summary of adjusted Cox proportional hazard analysis for cardiovascular events in the study.

Over a mean follow-up duration was 4.5 years for the scrub typhus group and 4.6 years for the comparison group, 138 participants died, and there were 45 cases of AMI, 56 cases of HFH, 27 hemorrhagic strokes, 63 ischemic strokes, 38 new-onset cases of AF, 9 aortic aneurysms or dissections, 11 cases of VTE, and 25 CV deaths. Table 2 presents the incidence rates of pre-specified CV events between the two groups during the study period. Patients with scrub typhus had higher rates of HFH and new-onset AF compared with the control group (3.62 vs. 1.84 and 2.63 vs. 1.06 per 1000 person-years, respectively), with rate ratios of 1.92 (95% CI: 1.10–3.34) and 2.39 (95% CI: 1.18–4.83), respectively. Cox regression analysis, adjusted for age, gender, and comorbidities, showed that the HR for HFH and new-onset AF in patients with scrub typhus compared to the control individuals were 1.97 (95% CI: 1.13–3.42, *p *= 0.016) and 2.48 (95% CI: 1.23–5.0, *p *= 0.011), respectively. The rates of AMI, hemorrhagic stroke, ischemic stroke, aneurysm or dissection of aorta, and CV death did not differ significantly between the two groups. When considering all major CV events combined (AMI, HFH, hemorrhagic or ischemic stroke, new-onset AF, aortic aneurysm or dissection, VTE, and CV death), a higher incidence was observed in the scrub typhus cohort than in the control group, with an aHR of 1.43 (95% CI: 1.08–1.91, *p *=  0.013). The cumulative incidence of each CV outcome, as determined by the Kaplan-Meier method, is displayed in Figures 2–4. Furthermore, subgroup analysis stratified by age, gender, and comorbid conditions showed that the impact of scrub typhus infection on subsequent CV risk remained consistent (Table 3).

**Table 2.** Cardiovascular outcomes during 5-year follow-up.

	*N*	Events	Total follow up	Incidence rate (/1000 person-years)	Incidence rate ratio (95% CI)	*P* value	Adjusted HR (95% CI)	*P* value
AMI								
Case	2264	25	10265.39	2.44	1.26 (0.70–2.27)	0.441	1.26 (0.70–2.28)	0.434
Control	2264	20	10346.89	1.93				
HFH								
Case	2264	37	10233.68	3.62	1.92 (1.10–3.34)	0.022	1.97 (1.13–3.42)	0.016
Control	2264	19	10350.28	1.84				
Hemorrhagic stroke								
Case	2264	13	10309.24	1.26	0.93 (0.44–1.99)	0.860	0.94 (0.44–1.99)	0.866
Control	2264	14	10374.29	1.35				
Ischemic stroke								
Case	2264	29	10275.01	2.82	0.86 (0.52–1.41)	0.549	0.86 (0.53–1.42)	0.557
Control	2264	34	10351.87	3.28				
New onset AF								
Case	2264	27	10264.46	2.63	2.39 (1.18–4.83)	0.016	2.48 (1.23–5)	0.011
Control	2264	11	10369.63	1.06				
Aneurysm and dissection of aorta								
Case	2264	3	10308.48	0.29	0.50 (0.13–2.01)	0.332	0.51 (0.13–2.02)	0.334
Control	2264	6	10377.78	0.58				
VTE								
Case	2264	6	10305.39	0.58	1.21 (0.37–3.96)	0.758	1.21 (0.37–3.97)	0.751
Control	2264	5	10376.79	0.48				
Cardiovascular death								
Case	2264	14	10315.02	1.36	1.28 (0.58–2.82)	0.539	1.28 (0.58–2.83)	0.536
Control	2264	11	10381.49	1.06				
All-cause death								
Case	2264	69	10315.02	6.69	1.01 (0.72–1.41)	0.970	1.01 (0.72–1.41)	0.959
Control	2264	69	10381.49	6.65				
All adverse cardiovascular events								
Case	2264	114	10110.61	11.28	1.41 (1.06–1.87)	0.019	1.43 (1.08–1.91)	0.013
Control	2264	81	10282.64	7.88				

AF: atrial fibrillation; AMI: acute myocardial infarction; HFH: heart failure hospitalization; HR: hazard ratio; VTE: venous thromboembolism.

All adverse cardiovascular events: AMI, HFH, hemorrhagic stroke, ischemic stroke, new onset AF, aneurysm and dissection of aorta, VTE, and cardiovascular death.

**Table 3.** Subgroup analysis for the risk of all adverse cardiovascular events.

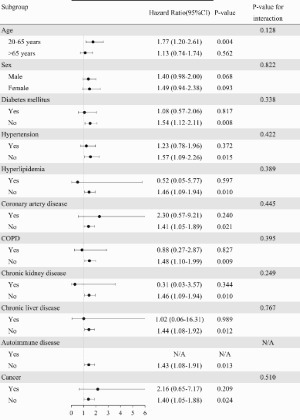				

## Discussion

This nationwide longitudinal observational study evaluates the long-term CV effects in patients infected with scrub typhus. Our findings revealed a 1.97-fold and 2.48-fold increased incidence of HFH and new-onset AF, respectively, in post-infection patients compared to a matched population (aHR 1.97, 95% CI: 1.13–3.42; aHR 2.48, 95% CI: 1.23–5.0). Additionally, a heightened risk for all major CV events was observed during the five-year follow-up (aHR 1.43, 95% CI: 1.08–1.91). Other cardiovascular events, such as AMI, cerebrovascular accidents, aortic diseases, VTE, and all-cause mortality did not significantly differ between patients with or without previous scrub typhus infection.

### Pathogenesis of scrub typhus

Our current understanding of the molecular pathogenesis and immune responses associated with scrub typhus is limited. In murine models of intradermal infection, *Orientia tsutsugamushi* evades autophagic elimination, replicating within mononuclear phagocytes and endothelial cells, spreading from the skin inoculation site, regional lymph nodes to target organs, leading to organ-specific pathology [15]. Severe infections in mice have highlighted a robust Th1/CD8-mediated immune response with suppressed Th2/regulatory T-cell markers, accompanied with loss of vascular endothelial integrity [16]. It is implicative of that dysregulated immune responses may contribute to tissue damage and organ dysfunction rather than direct bacterial cytotoxicity [17]. However, what are the critical elements triggering the host inappropriate immune responses and how to restore the balance avoiding serious complications still need to be investigated.

### Risks of heart failure hospitalization in scrub typhus

Heart failure syndrome resulting from acute scrub typhus infection may have been underestimated in previous literature [18]. Prospective cohort studies assessing cardiac manifestations of scrub typhus through echocardiography and cardiac biomarkers have demonstrated significant cardiac involvement: 30.9%–42.8% of patients exhibited a reduced ejection fraction, and 61.7%–72.8% showed elevated levels of troponin T or creatine kinase-muscle/brain isoenzyme [10,11]. These elevated biomarkers correlated with the degree of systolic dysfunction, suggesting that cardiac injury and systolic dysfunction are not uncommon in acute scrub typhus infection.

Although the immune response aims to eradicate infected cells, dysregulated inflammation can lead to structural and functional cardiomyocyte abnormalities, resulting in contractility failure, chamber remodeling, or conduction disturbances. Furthermore, *Orientia tsutsugamushi* can cause persistent or latent infections in some patients following acute illness, leading to continuous macrophage activation, which hinders inflammatory resolution and delays tissue healing [19].

Our cohort study observed an increased risk of HFH in patients during the 4–5 years after contracting scrub typhus, a finding not previously reported. Neurohormonal blockade is critical in addressing the pathophysiological dysregulation associated with low cardiac output in patients with heart failure. There is robust evidence supporting the long-term survival benefits of neurohormonal blockade in these patients. Early treatment with doxycycline may reduce the risk of future CV events in scrub typhus patients by effectively eliminating *Orientia tsutsugamushi* and mitigating the inflammatory cascade that contributes to CV complications. In cases where scrub typhus leads to heart failure, as inflammatory cardiomyopathy, early targeted therapy to address the underlying infection and appropriate anti-inflammatory treatments may help minimize inflammation, tissue damage, and cardiac dysfunction. Although our study did not directly evaluate the impact of early doxycycline administration or specific interventions for scrub typhus-associated heart failure, the rationale for these approaches is strong. Unfortunately, there is currently no direct evidence or established guidelines for managing heart failure in this specific clinical scenario. Further research is needed to determine the optimal therapeutic strategies in such cases.

This observation highlights two critical clinical implications: First, early diagnosis of scrub typhus and prompt management of associated complications, such as heart failure, are essential. Treatment with doxycycline to eliminate *Orientia tsutsugamushi* and the use of neurohormonal blockade to address heart failure-related pathophysiological dysregulation could improve patient outcomes. Early interventions may help reverse pathological cardiac remodeling and enhance long-term prognosis. Second, post-infection cardiac surveillance should be prioritized, particularly in patients presenting with symptoms or signs suggestive of cardiac involvement. Routine cardiac monitoring, including echocardiography and ECG before discharge, is a compelling approach given the frequent cardiac involvement in scrub typhus. Such monitoring could facilitate the early detection of subclinical cardiac abnormalities, enabling timely intervention. Additionally, reducing the time between symptom onset and accurate diagnosis is crucial to preventing excessive inflammatory responses and mitigating cardiac tissue damage. However, practical considerations such as resource allocation and cost-effectiveness should guide its implementation, potentially by targeting high-risk patients identified through clinical assessment and biomarkers.

This approach underscores the importance of heightened awareness among healthcare providers and the availability of reliable diagnostic methods to ensure effective management of scrub typhus-related cardiac complications [20].

### Risk of atherosclerotic cardiovascular disease in scrub typhus

Chronic persistent inflammation is a recognized pathophysiologic mechanism underlying atherosclerotic cardiovascular diseases [21]. Microbial products can activate the innate immune system through toll-like receptors, triggering a cascade of inflammatory reactions within vascular atheromas [22]. The presence of *Orientia tsutsugamushi*, leads to rickettsemia and can replicate or persist within the vascular endothelium and immune cells, prompting concerns regarding its role in atherosclerotic events. Notably, transient ischemic attacks and symptomatic CAD have been documented to occur 4–6 months post-infection, with the persistence of viable *O. tsutsugamushi* confirmed by cell culture and nucleotide sequencing [19].

Contrasting evidence from a healthcare claims database indicated that patients with scrub typhus had a 60% increased risk of subsequent AMI, although not cerebrovascular accidents, compared to the control population [23]. Our study, however, did not find a significant increase in the risk of ischemic stroke or AMI in patients with scrub typhus. Such discrepancies could be attributable to differences in the patient cohorts selected, the prevalent strains of the bacterium, and the duration of follow-up between studies. It is plausible that our younger patient cohort, which had no prior CV events or serious complications and was observed over a shorter duration (4.5 years), may have been less likely to develop major atherosclerotic events than those in studies with longer follow-ups. The current state of evidence does not allow for a definitive conclusion regarding the relationship between scrub typhus and atherosclerotic events. Prospective studies with deliberate design are warranted to establish a clear understanding of this potential association.

### New onset atrial fibrillation in scrub typhus

AF is common arrhythmia in the elderly and in individuals with CV comorbidities, characterized by rapid and irregular electrical activities originating from multiple atrial foci. The pathogenesis of AF typically involves ectopic atrial activity, particularly from the muscular sleeves of the pulmonary vein ostia, as well as changes in the structural and electrical properties of the atria, which may include multiple re-entrant circuits and an associated inflammatory process [24]. Inflammatory cascades can induce atrial myocyte apoptosis, calcium overload, tissue fibrosis, and sarcomeric ion exchanger dysfunction, all of which contribute to the initiation and maintenance of AF [25,26].

Within our cohort, there was an observed increase in the risk of new onset AF among patients who had suffered from scrub typhus. The likely underpinning for this finding is the acute or chronic inflammatory stimuli from the infection leading to “atrial myocarditis” and subsequent atrial remodeling. The clinical significance of AF in the context of scrub typhus has been previously underscored in a cohort study where new-onset AF was linked to increased risks of short-term mortality and acute heart failure [27]. Furthermore, the presence of paroxysmal AF in patients with scrub typhus was reported as a predictor of an underlying acute myocarditis [28]. The clinical course of acute myocarditis can vary widely. While some patients experience mild, self-limiting symptoms, others may progress to develop refractory cardiogenic shock, necessitating mechanical circulatory support. Therefore, early detection and appropriate management of myocarditis associated with scrub typhus are crucial for the prognosis. New-onset AF may serve as a readily identifiable and valuable indicator, alerting clinicians to the potential for serious cardiac complications.

### Study limitations

This study has several limitations inherent to its retrospective, observational design. Retrospective analyses may be prone to confounding factors related to patient characteristics influencing infection susceptibility. Although we minimized confounding by excluding individuals with prior CV events and matching controls based on age, gender, and comorbidities, residual unmeasured biases may persist.

A key limitation is the absence of residential area as a matching criterion. Scrub typhus cases predominantly occur in rural areas with limited healthcare access, while controls may more frequently reside in urban areas. This imbalance could introduce potential confounding, as urban exposures such as sedentary lifestyles and high fat foods, contribute to an increased risk of ischemic heart disease and stroke [29–31]. Conversely, urban areas often provide better access to healthcare services and advanced treatments, which can improve outcomes for CV disease management. However, despite this limitation, our findings demonstrated a robust association between scrub typhus infection and increased risks of HFH and new-onset AF, suggesting that the relationship is likely not solely attributable to differences in residence.

CV outcomes were determined using discharge diagnostic codes, limiting access to finer clinical details like echocardiographic or electrocardiographic findings, limiting provide the potential impact of disease severity on the observed outcomes. Additionally, the database does not differentiate between *Orientia* species. Since *O. tsutsugamushi* is the predominant species in Taiwan, our findings primarily apply to infections caused by this species. Finally, we did not adjust for the use of cardiovascular preventive medications, which may influence CV event risks, but individuals included in the study were free of major CV events at baseline.

Despite these limitations, this large-scale nationwide study provides valuable insights into the association between scrub typhus and CV outcomes. Future prospective studies are needed to confirm these findings and further clarify the cardiovascular implications of scrub typhus infection.

## Conclusions

This retrospective cohort study observed an increased risk of subsequent CV events following scrub typhus infection, particularly HFH and new-onset AF. The involvement of inflammatory cascades during infection may lead to subclinical, yet progressive, CV system abnormalities that manifest over time. These findings highlight the need for heightened CV risk awareness in patients with a history of scrub typhus and the consideration of cardiac surveillance when clinically indicated.

## Supplementary Material

Supplementary.docx
