# Supplementary material for: Beyond the infection: mapping the risk of cardiovascular events post-scrub typhus in a nationwide cohort study
Source: Emerg Microbes Infect. 2025 Feb 13;14(1):2467766. doi: 10.1080/22221751.2025.2467766 (PMC11843642; doi:10.1080/22221751.2025.2467766)
Supplement: Supplementary.docx [file TEMI_A_2467766_SM3191.docx]

**Supplemental Table 1. ICD-9-CM and ICD-10-CM codes for disease and diagnosis used in this study**

|  | **ICD-9-CM** | **ICD-10-CM** | Notes |
| --- | --- | --- | --- |
| **Scrub typhus** | As designated by Notifiable Disease Dataset of Confirmed Cases – Disease Prevention Database (NDDCC) | | |
| **Comorbid conditions** |  |  |  |
| Diabetes mellitus | 250.x | E08.x, E09.x, E10.x, E11.x, E12.x, E13.x | Outpatient diagnosis or discharge diagnosis |
| Hypertension | 401.x, 402.x, 403.x, 404.x, 405.x | I10.x, I11.x, I12.x, I13.x, I15.x, N26.2 |  |
| Hyperlipidemia | 272.0, 272.1, 272.2 | E78.0, E78.1, E78.2 |  |
| Coronary artery disease | 414.x | I25.x |  |
| Chronic obstructive pulmonary disease | 416.8, 416.9, 490, 491, 492, 493, 494, 495, 496, 500, 501, 502, 503, 504, 505, 506.4, 508.1, 508.8 | I27.8, I27.9, J40.x, J41.x, J42.x, J43.x, J44.x, J45.x, J46.x, J47.x, J60.x, J61.x, J62.x, J63.x, J64.x, J65.x, J66.x, J67.x, J68.4, J70.1, J70.3 |  |
| Chronic kidney disease | 585.x | N18.x |  |
| Chronic liver disease | 571.x | K70.x, K73.x, K74.x |  |
| Autoimmune disease | Registry for Catastrophic Illness Patients | | |
| Cancer | 140.x~199.x | Cxx.x | Registry for Catastrophic Illness Patients |
| **Outcomes** |  |  |  |
| Acute myocardial infarction | 410.x, 411.x | I20.x, I21.x, I22.x, I24.x | Discharge diagnosis or cause of death data |
| Heart Failure hospitalization | 428.x | I50.x | Discharge diagnosis or cause of death data |
| Hemorrhagic Stroke | 430.x, 431.x, 432.x | I60.x, I61.x, I62.x | Discharge diagnosis or cause of death data |
| Ischemic Stroke | 433.x, 434.x, 435.x, 436.x, 437.x | I63.x, I65.x, I66.x, I67.x, I68.x, G45.x, G46.x | Discharge diagnosis or cause of death data |
| New onset atrial fibrillation | 427.31 | I48.0, I48.1, I48.2, I48.91 | Discharge diagnosis or cause of death data |
| Aneurysm and Dissection of aorta | 093.0, 094.87, 362.14, 414.11, 417.1, 437.3, 441.x, 442.x | A52.01, A52.19, H35.041, H35.042, H35.043, H35.049, I25.41, I25.42, I28.1, I67.0, I67.1, I71.x, I72.x, I77.7, I77.819, I79.x | Discharge diagnosis or cause of death data |
| Venous thromboembolism | VTE codes were categorized as pulmonary embolism (ICD-9 code 415.1x), lower extremity DVT (451.1x, 451.2, 451.81, 453.4x, 453.5x), upper extremity DVT (451.83, 451.84, 451.89, 453.72, 453.73, 453.74, 453.75, 453.76, 453.77, 453.82, 453.83, 453.84, 453.85, 453.86, 453.87), and other venous thrombosis (451, 451.9, 452, 453, 453.0, 453.1, 453.2, 453.3, 453.79, 453.8, 453.89, 453.9). | I80.0-I80.3, I80.8-I80.9, I82.9, O22.2 – O22.3, O87.0 – O87.1, I26.0, and I26.9. | Outpatient diagnosis or discharge diagnosis |
| Cardiovascular death |  | I0x.x, I2x.x, I3x.x, I4x.x, I5x.x, I6x.x, I7x.x, I8x.x, I9x.x | Cause of death data |
| All-cause death |  |  | Cause Of Death Data |
| All adverse cardiovascular events | Acute myocardial infarction or Heart Failure or Hemorrhagic stroke or Ischemic stroke or Atrial fibrillation or Aneurysm and dissection of aorta or Venous thromboembolism or Cardiovascular death | | |
